# A Nighttime Telemedicine and Medication Delivery Service to Avert Pediatric Emergencies in Haiti: An Exploratory Cost-Effectiveness Analysis

**DOI:** 10.4269/ajtmh.21-1068

**Published:** 2022-02-21

**Authors:** Katelyn E. Flaherty, Molly B. Klarman, Youseline Cajusma, Justin Schon, Lerby Exantus, Valery M. Beau de Rochars, Chantale Baril, Torben K. Becker, Eric J. Nelson

**Affiliations:** ^1^ Departments of Pediatrics and Environmental and Global Health, University of Florida, Gainesville, Florida;; ^2^ Department of Emergency Medicine, University of Florida, Gainesville, Florida;; ^3^ College of Arts and Sciences, College of William and Mary, Williamsburg, Virginia;; ^4^ State University of Haiti, Port-au-Prince, Haiti;; ^5^ Department of Health Services Research, Management and Policy, University of Florida, Gainesville, Florida

## Abstract

We sought to compare the costs of a nighttime pediatric telemedicine and medication delivery service per disability-adjusted life year (DALY) averted to the costs of current hospital emergency care per DALY averted from a societal perspective. We studied a nighttime pediatric telemedicine and medication delivery service and hospital emergency care in a semi-urban and rural region of Haiti. Costs of the two services were enumerated to represent the financial investments of both providers and patients. DALYs averted were calculated to represent the “years lives lost” and “years lost to disability” from diarrheal, respiratory, and skin (bacterial and scabies etiologies) disease among children from 0 to 9 years old. The incremental cost-effectiveness ratio was estimated and compared with the per capita gross domestic product (GDP) of Haiti ($1,177). Cost-effectiveness was defined as an incremental cost-effectiveness ratio less than three times the per capita GDP of Haiti ($3,531). The total costs of the nighttime telemedicine and medication delivery service and hospital emergency care to society were $317,898 per year and $89,392 per year, respectively. The DALYs averted by the service and hospital emergency care were 199.76 and 22.37, respectively. Correspondingly, the incremental cost-effectiveness ratio is estimated at $1,288 signifying that the service costs an additional $1,288 to avert one additional DALY. A scaled nighttime pediatric telemedicine and medication delivery service is likely a cost-effective alternative to hospital emergency care for pre-emergency pediatric conditions in Haiti, and possibly in similar lower-middle-income countries.

## INTRODUCTION

Respiratory infection and diarrheal disease are the leading causes of pediatric mortality for children aged between 1 month and 5 years globally and resulted in over 1 million deaths in 2019. ^1^ Well-established low-cost treatments exist for both acute respiratory infections and diarrheal diseases. ^2^^,^ ^3^ Oral amoxicillin for bacterial pneumonia can reduce mortality by 32% ^4^ and treatment of diarrhea with oral rehydration solution and zinc can reduce mortality by 93% ^5^ and 23%, ^6^ respectively. However, these treatments are most effective when administered soon after symptoms start, which is difficult when healthcare access is limited, particularly at night. Delayed treatment, especially in children, can result in rapid progression to an emergent state. ^7^ In these settings, emergency care is considerably more difficult to access and more expensive than pre-emergency care. ^8^^,^ ^9^ More than half of the 5.3 million early childhood deaths in 2018 were considered preventable with basic healthcare. ^10^

In Haiti, the leading causes of death among children younger than 5 years are acute respiratory infections, prematurity, and diarrhea. ^11^ About 62% of the population lives below the international poverty level of $1.25/day. ^12^ Government healthcare spending per capita is $8, below the low income country average of $9. ^13^ Over one-third of this funding is spent at the hospital level, leading to an over reliance on emergency services. ^14^ Only 23% of the total population and 5% of the rural population have access to quality primary care. ^11^ Child health and wellness is substandard, with Haiti ranking 151 out of 180 countries on the Flourishing Index. ^12^ The under-five mortality rate is 65 per 1,000 live births compared with 39 per 1,000 live births globally. ^15^ Respiratory infection and diarrheal disease are major contributors to this burden, accounting for over half of hospitalizations and over a third of deaths of hospitalized children. ^16^

Improving access to healthcare is one of the highest global health priorities set by the sustainable development goals (SDG). ^17^^,^ ^18^ SDG 3.8 seeks to “achieve universal health coverage,” however, progress is insufficient to reach this target by 2030. ^18^^,^ ^19^ Low- and middle-income countries (LMIC) are the furthest off this target. ^20^ Innovative, cost-effective approaches are needed to overcome persistent as well as emerging barriers to improve and sustain healthcare access in LMIC.

Telemedicine and medication delivery services are one such approach. The objective of the study herein was to conduct a cost-effectiveness analysis of a scaled telemedicine and delivery service model. We determined and compared the cost of the nighttime pre-emergency pediatric service per disability-adjusted life year (DALY) averted to the cost of hospital emergency care per DALY averted.

## METHODS

### Ethics statement.

Ethical approvals were obtained from the Comité National de Bioéthique (National Bioethics Committee of Haiti) and the University of Florida Institutional Review Board for participants in INACT-1 (1718-35; IRB201703246) and INACT-2 (1920-42; IRB201802920). For both studies, participants provided written informed consent/assent. For INACT-2, a waiver of documentation of written consent was obtained for those without a household delivery.

### Foundational work.

To better understand the barriers to pediatric healthcare in Haiti, we conducted a needs assessment to identify differences in household healthcare seeking intention (“would do”) and behavior (“did do”) ^21^; this initiative was part of the Improving Nighttime Access to Care and Treatment Study (INACT-1). ^21^ In this needs assessment, households expressed an intention to seek care from a provider within conventional networks, but, because of cost, distance, and nighttime hours, sought care from disconnected providers near to the household in practice. INACT-1 revealed that a pediatric telemedicine and medication delivery service, known as MotoMeds, might provide an innovative solution to one aspect of the crisis of limited access to pediatric healthcare.

**Figure 1.**
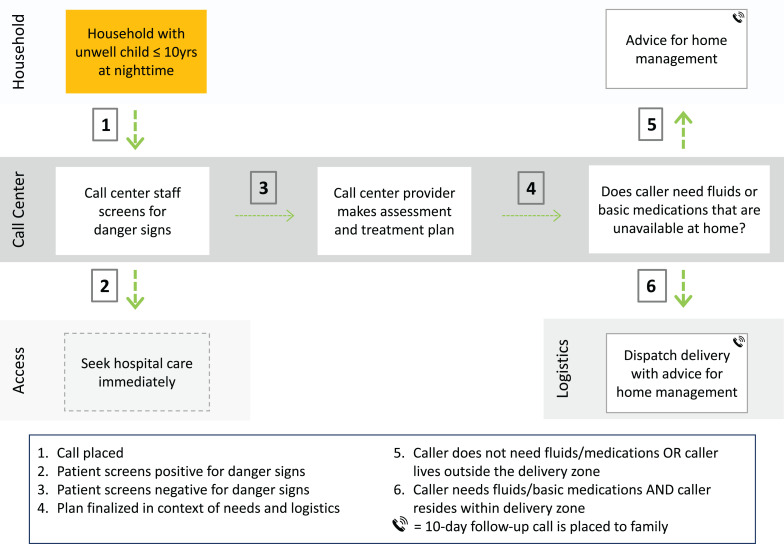
Scaled MotoMeds workflow. This figure appears in color at www.ajtmh.org.

The service was launched in Gressier, Haiti, as a pre-pilot (INACT-2) in September 2019. Families with children experiencing pre-emergent medical problems at night called the call center. A nurse with physician oversight used a clinical decision-support tool adapted from the WHO Integrated Management of Childhood Illness guidelines ^22^^,^ ^23^ to triage, conduct an assessment, and generate a plan and logistics system to enable household medication delivery. Patients identified with life-threatening conditions were referred for emergency care. During INACT-2, call center nurses were dispatched to all households to confirm that call center findings matched in-person findings (Figure 1). Respiratory illness, diarrhea, and skin problems (bacterial infections, scabies infestations) were common, and the median time to delivery was 1 hour 20 minutes. At 10 days, 94% of parents reported the problem resolved/had improved.

### Study design.

The cost of the intervention, the scaled telemedicine and medication delivery service, per DALY averted was modeled and compared with the cost of existing healthcare options, hospital emergency care, per DALY averted from a societal perspective. The rationale was that the service and hospital emergency care are the only accessible healthcare options at night. Costs represent the financial investments of both providers and patients. The incremental cost-effectiveness ratio describing additional costs of the telemedicine and medication delivery service per DALY averted was estimated and compared with the per capita GDP of Haiti ($1,177 in 2020). ^24^^,^ ^25^

### Setting and participants.

In 2019, the MotoMeds service was launched in Gressier, Haiti (INACT-2), a mountainous commune of the Port-au-Prince Arrondissement with a population of 38,092. ^26^ A 78 sq km delivery zone extending 5 km out from the call center was established, encompassing approximately 89% of the population. MotoMeds will expand to the similar but geographically distinct communes of Leogane and Jacmel (Supplemental material 1) which have populations of 208,799 and 195,674, respectively. ^26^ Analyses herein used data from the INACT-1 needs assessment and INACT-2 pre-pilot to evaluate the cost-effectiveness of the scaled service in Gressier, Leogane, and Jacmel (Supplemental material 1). ^27^^,^ ^28^ The “scaled” model for MotoMeds restricts household visits to mainly moderate cases and few mild cases; severe cases bypass MotoMeds for hospital referral.

### Population and utilization estimations.

Delivery zones were delineated in each location based on distance and terrain. Inhabitants within these zones were referred to as the “accessible population.” The delivery zone for Gressier remained unchanged from INACT-2 and encompasses the second and third communal sections. Accessible populations of Leogane encompass the first, second, and third communal sections while accessible populations in Jacmel encompass the urban and half of the rural communal sections of Bas Cap Rouge, Ravine Normande, and Gaillard. Populations were extrapolated from 2015 data with an estimated 4.50% growth rate to 2020. ^29^ The service coverage and patient load were calculated through extrapolation of INACT-2 data to account for increased advertising and awareness of the service and expansion to accessible regions of Leogane and Jacmel. Hospital emergency care utilization and patient load were estimated from INACT-1 data and 2016 Demographic and Health Surveys (DHS) data relating to the proportion of the population that seeks hospital level care. INACT-1 found that families sought care for 32% of pediatric health events. ^21^ The 2016 DHS in Haiti found that children under 5 sought care for 40.4% of acute respiratory infections and 32.9% of diarrheal cases. ^30^ These figures were averaged to estimate 35.1% of families with a sick child seek care. The INACT-1 found that 8% of care-seekers seek hospital care. ^21^ DHS data were used to determine the proportion of the accessible population under 1 year (2.2%), from 1 to 4 years (7.5%), and 5 to 9 years (11.3%). ^30^ The age distribution was assumed to be geographically uniform across Haiti.

### Cost estimation.

All costs were converted from Haitian gourdes to US dollars ($) at a rate of 1 US dollar to 90.15 Haitian gourdes (24-month average July 2019 to June 2021). ^31^

#### Telemedicine and delivery service.

**Figure 2.**
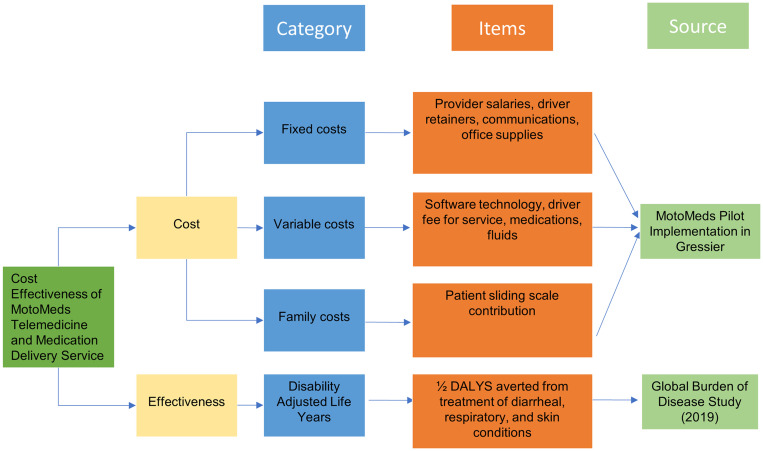
Costs and effects of MotoMeds telemedicine and medication delivery service. This figure appears in color at www.ajtmh.org.

Fixed, variable, and family costs were summed to determine the societal cost of the telemedicine and medication delivery service. Costs represented the operational budget and did not include start-up expenses. Primary data were obtained from 16 months of MotoMeds operations in Gressier (INACT-2). Fixed costs refer to costs that did not vary according to patient volume, whereas variable costs refer to costs that did vary according to patient volume. Family costs refer to the amount paid by patient families for the service (Figure 2).

Fixed costs: The supervisor, study physician(s), and on-call physician(s) were assumed to be paid a fixed monthly rate. The call center nurses were assumed to be compensated per shift worked. In INACT-2, Gressier drivers were paid a daily retainer; however, this was not included in the compensation scheme for Jacmel and Leogane given the size of the communities and corresponding number of expected deliveries for which drivers will be compensated. For the pre-pilot, the service used two call center nurses and two drivers per night. To serve Gressier, Leogane, and Jacmel, the service at scale was expected to use four call center nurses, five on-call nurses and 14 drivers per night. Human resource administrative fees were estimated to be between 8–10%. Fixed costs also captured monthly operational costs (e.g., office rent, internet, and phone plans); drivers and on-call nurses were determined to receive a set amount of cellular phone credits monthly.

Variable costs: Variable costs included driver delivery payment, medications, and fluids. They were calculated per patient and multiplied by the expected number of patients per year. Half of deliveries were estimated to require two drivers and 20% of deliveries were estimated to require a nurse visit due to driver safety and patient severity, respectively. Variable costs also captured software technologies used by the telemedicine and medication delivery service for call intake (Twilio) ^32^ and driver dispatch (Beacon by TrekMedics). ^33^

Family costs: Costs to families refer to the amount families pay to use the telemedicine and medication delivery service. Under the service model, families are asked to pay what they are able to on a sliding scale starting from 500 gourdes ($5.78) down to zero gourdes. During INACT-2, 30% of families paid in full and 25% paid in part (250 gourdes, $2.89). ^21^ We conservatively assumed the same ratio for these analyses.

Based on INACT-1 findings, 1% of telemedicine and medication delivery service patients are expected to later require hospital emergency care. Therefore, hospital emergency care costs were added to the cost of service for 1% of expected patients.

#### Hospital emergency medicine.

**Figure 3.**
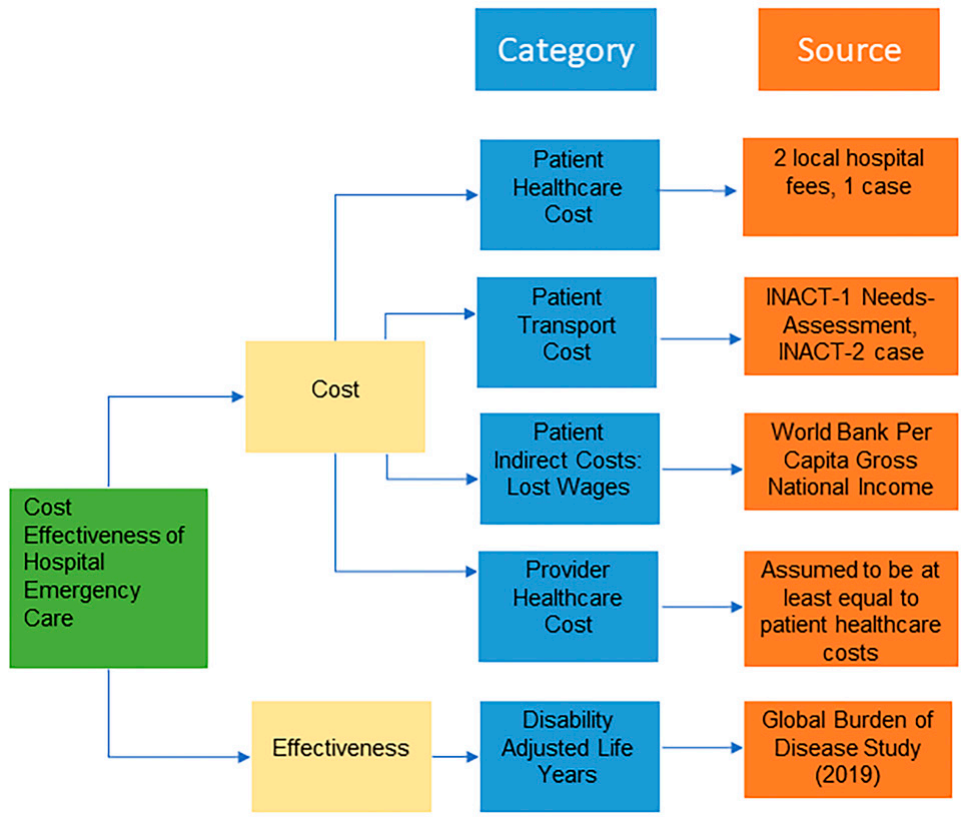
Costs and effects of hospital emergency care. This figure appears in color at www.ajtmh.org.

Patient and provider/institutional costs were summed to determine the societal cost of hospital emergency care (Figure 3). Primary data were obtained from fees at 2 main local hospitals, one private and the other public/charity, and INACT-1 data. Patient costs captured the amount families pay for pediatric care for diarrheal, respiratory, or bacterial skin disease. Patient costs associated with diarrheal disease were calculated from the costs of admission, a 2-day hospital stay, urine and stool testing (part of the local standard of practice), amoxicillin, IV fluids and oral rehydration solution. Patient costs associated with respiratory disease were calculated from the costs of admission, a 2-day hospital stay, oxygen, a hemogram including complete blood count, amoxicillin, and an X-ray. Patient costs associated with bacterial skin disease were determined from a single case from INACT-2 in which a pediatric patient with a bacterial skin infection required a 2-day hospital stay. Patient healthcare costs were calculated as a weighted average of the costs to treat diarrheal, respiratory, and skin disease based on condition prevalence in Haiti. ^34^ Patient costs additionally captured the amount families pay for transportation to the hospital. Patient transportation costs were calculated as a weighted average of reported transportation costs from INACT-1 data and an INACT-2 bacterial skin infection case. Patient indirect costs accounted for 2 days of lost wages by a caretaker. Daily wages were estimated from the mean per capita gross national income. We were not able to obtain provider/institutional costs, likely due to the absence of specific costing data at local hospitals. ^35^ As a result, provider costs were conservatively assumed to be equal to or greater than the direct healthcare patient costs for hospital emergency care.

### Effect estimation.

#### Telemedicine delivery service.

The key outcome indicator was the DALYs averted by treatment of pediatric pre-emergency conditions. The total number of DALYs averted by the scaled telemedicine and medication delivery service was calculated using data from the 2019 Global Burden of Disease (GBD) Study ^1^ to determine the years of life lost due to premature mortality (YLL) and years of healthy life lost due to disability (YLD) for the 4 conditions most commonly treated by the service: respiratory infection, diarrheal disease, bacterial skin infection, and scabies skin infestation.

The total number of DALYs averted by the telemedicine and medication delivery service within the accessible population who are likely to use the service for children under ten years old was estimated using the following formula: DALY = YLL + YLD. YLL was estimated by multiplying the sum of half of the mortality rates associated with respiratory infection, diarrheal disease, bacterial skin infection, and scabies skin infestation by the life expectancy for children under one year old, one to 4 years old and 5 to 9 years old, which was 63.71 years, 61.21 years and 56.48 years, respectively. ^36^ Mortality rates were halved to account for the service operating at nighttime alone. YLD was estimated by summing half the prevalence of respiratory infection, diarrheal disease, bacterial skin infection, and scabies skin infestation multiplied by the corresponding disability weights for children aged under one year old, one to 4 years old and 5 to 9 years old per the GBD Study. ^1^ Prevalence rates were halved to account for the telemedicine and medication delivery service operating at nighttime only. DALY estimates were calculated for the proportion of the accessible population likely to use the service (see utilization estimates). DALY estimates were discounted at 3% in accordance with global health convention. ^37^

#### Hospital emergency medicine.

The total number of DALYs averted by patient utilization of hospital emergency care was calculated for the same four conditions. In YLL and YLD calculations, the full mortality and prevalence estimates were used as hospital emergency care is available at all times of day and night. DALY estimates were calculated for the proportion of the accessible population likely to use hospital emergency care (see utilization estimates). DALY estimates were again discounted at 3%.

### Cost-effectiveness estimation.

The incremental cost-effectiveness ratio was estimated using the following formula: (Cost of Telemedicine and Medication Delivery Service − Cost of Hospital Emergency Care) / (DALYs Averted by Telemedicine and Medication Delivery Service – DALYs Averted by Hospital Emergency Care). In accordance with WHO recommendations, ^38^ cost-effectiveness is defined herein as when the incremental cost-effectiveness ratio of an intervention is less than three times ($3,531) the per capita GDP of Haiti ($1,177 in 2020). ^25^

### Sensitivity analysis.

Univariate sensitivity analysis was performed to evaluate how uncertainty of the parameter estimates affects the incremental cost-effectiveness ratio. The following parameters were varied to low and high values: proportion of the population utilizing the telemedicine and medication delivery service (25–75%), proportion of the population utilizing hospital emergency care (0–5%), cost of hospital emergency care per patient ($100-180), cost of hospital transport per patient ($0–$10), cost of two days of lost wages ($0–$14) and discounting rate (0–6%). The number of patients served by the telemedicine and medication delivery service was varied in accordance with the proportion of the population utilizing the service. Likewise, the number of patients served by hospital emergency care was varied in correspondence with the proportion of the population utilizing hospital emergency care.

### Data analysis.

Data analysis was conducted in Microsoft Excel and R Version 4.0.3. ^39^ DALY calculations were performed within the WHO Human Services Handbook Excel template for DALY calculation. To estimate the cost and effect of the telemedicine and medication delivery service at-scale, we first assumed that diarrheal disease, respiratory infections, bacterial skin infections, and scabies skin infestation will remain the conditions most commonly treated by future iterations of the service. Additionally, it was assumed that the service will serve similar population proportions in Gressier, Leogane, and Jacmel. Furthermore, it was assumed that the exchange rate will remain relatively stable at the 24-month average. ^31^

## RESULTS

### Population and utilization estimations.

The accessible population is estimated to be 33,971 in Gressier, 173,838 in Leogane, and 65,630 in Jacmel. The total accessible population is estimated to be 273,440 with 57,422 children under 10 years of age. During the INACT-2 study that had minimal advertising, one to two patients per night were observed. We estimate approximately 25% of the accessible population of Gressier was aware of the telemedicine and medication delivery service given Fall 2020 outreach that revealed the percentage of persons approached in markets who were aware of the service to be modestly below 25%. In a scaled TDMS model, advertising efforts are expected to be doubled to increase the utilization to 50%. This is expected to result in four patients per night in Gressier, 18 patients per night in Leogane, and seven patients per night in Jacmel, in correspondence with the accessible populations of each location. Therefore, a scaled service model is anticipated to serve 29 patients per night and 10,585 patients per year. Given 35% of families with a sick child seek care and 8% of care-seekers seek hospital care, it is estimated that 2.8% of the accessible population will use hospital emergency care, 47.2% less than the telemedicine and medication delivery service, for a total of 296 cases per year (Supplemental material 2).

### Cost estimations.

#### Telemedicine and medication delivery service.

Fixed costs totaled $105,531, whereas variable costs totaled $155,447 for 10,585 patient encounters per year. Patient costs were estimated at $24,954. Thus, assuming 10,585 patient encounters per year by the telemedicine and medication delivery service, the total cost of the service to society is estimated at $285,932 per year (Table 1). Assuming 1% of patients require hospital emergency care follow-up, the cost of the service and emergency follow-up is estimated at $317,898 per year. The costliest component of the telemedicine and medication delivery service is staff compensation, specifically nurse and driver compensation.

**Table 1:** MotoMeds yearly societal costs

	Amount ($)	Notes
Fixed costs
Clinical staff	82,627	Physician and nurse salaries
Nonclinical staff	5,975	Director salary, driver retainers
Office	8,089	Rent, supplies
Communication	6,440	Phone, Internet
Advertising	2,400	Flyers, radio time
Variable costs
Driver	112,762	Compensation for deliveries with and without nurse
Treatment	33,449	Medications, fluids
Technology	9,236	Twilio, Beacon
Patient costs
Family payment	24,954	Family full or partial payment
**Total societal cost**	**285,932**	

#### Hospital emergency medicine.

The estimated patient cost of hospital emergency care for pneumonia was $201 at one local emergency center and $151 at another local emergency center for an average of $176. The estimated patient cost of hospital emergency care for diarrhea was $131 at one center and $60 at the other for an average of $95. The patient cost of hospital emergency care for a bacterial skin infection was $150 at one local emergency center. Therefore, when averaged using global burden of disease prevalence weights, the estimated patient cost of hospital emergency care is $145 per patient. ^1^ In INACT-1, 9 pediatric patients who sought hospital emergency care reported an average transportation cost of $4.13 (daytime versus nighttime was not known). ^21^ In INACT-2, a single patient reported a transportation cost of $8.87 at night. Using these figures, the weighted average estimate for hospital transportation cost is approximately $5 per patient. The 2019 per capita gross national income in Haiti was reported at $1,330/year ($3.64/day). ^40^ As a result, indirect costs include $7 in caregiver lost wages per patient for 2 days of hospital emergency care. Provider/institutional costs were conservatively assumed to be equal to the direct patient healthcare costs of $145 per patient. Assuming 296 patients access hospital emergency care per year, 2.8% of the accessible population, the total cost of hospital emergency care to society is $89,392 (Table 1).

### Effect estimations.

#### Telemedicine and medication delivery service.

Nighttime treatment of diarrheal diseases, respiratory infections, bacterial skin infections, and scabies skin infestations was found to avert 17.1 YLL and 28.6 YLD among children younger than 1 year old, 25.1 YLL and 68.2 YLD among children 1–4 years old, and 4.3 YLL and 56.4 YLD among children 5–9 years old. It was determined that the telemedicine and medication delivery service would avert 199.76 DALYs per year of operation.

#### Hospital emergency care.

Hospital emergency treatment of diarrheal diseases, respiratory infections, bacterial skin infections, and scabies skin infestations was found to avert 1.9 YLL and 3.2 YLD among children younger than 1 year old, 2.8 YLL and 7.6 YLD among children 1–4, and 0.5 YLL and 6.3 YLD among children 5–9. It was determined that hospital emergency care would avert 22.37 DALYs per year.

In the telemedicine and medication delivery service and hospital emergency care DALY calculations, treatment of respiratory infections was found to avert the most YLL in all age groups. Treatment of diarrheal disease was found to avert the most YLD in all age groups.

### Cost-effectiveness estimation.

The telemedicine and medication delivery service is estimated to cost society approximately $228,506 more than hospital emergency care yet averts an additional 177.39 DALYs per year (Table 2). Correspondingly, the incremental cost-effectiveness ratio is estimated at $1,288 signifying the service costs an additional $1,288 to avert one additional DALY. This value is under 2× the per capita GDP of Haiti, $1,177 in 2020, ^25^ therefore, the telemedicine and medication delivery service is considered cost effective by WHO standards. ^24^

**Table 2:** Comparison of telemedicine and medication delivery service and hospital emergency care by cost and DALYs averted

	Cost ($)	DALYs Averted
Telemedicine and medication delivery service	317,898	199.8
Hospital emergency care	89,392	22.4
Difference	228,506	177.4

### Sensitivity analysis.

**Figure 4.**
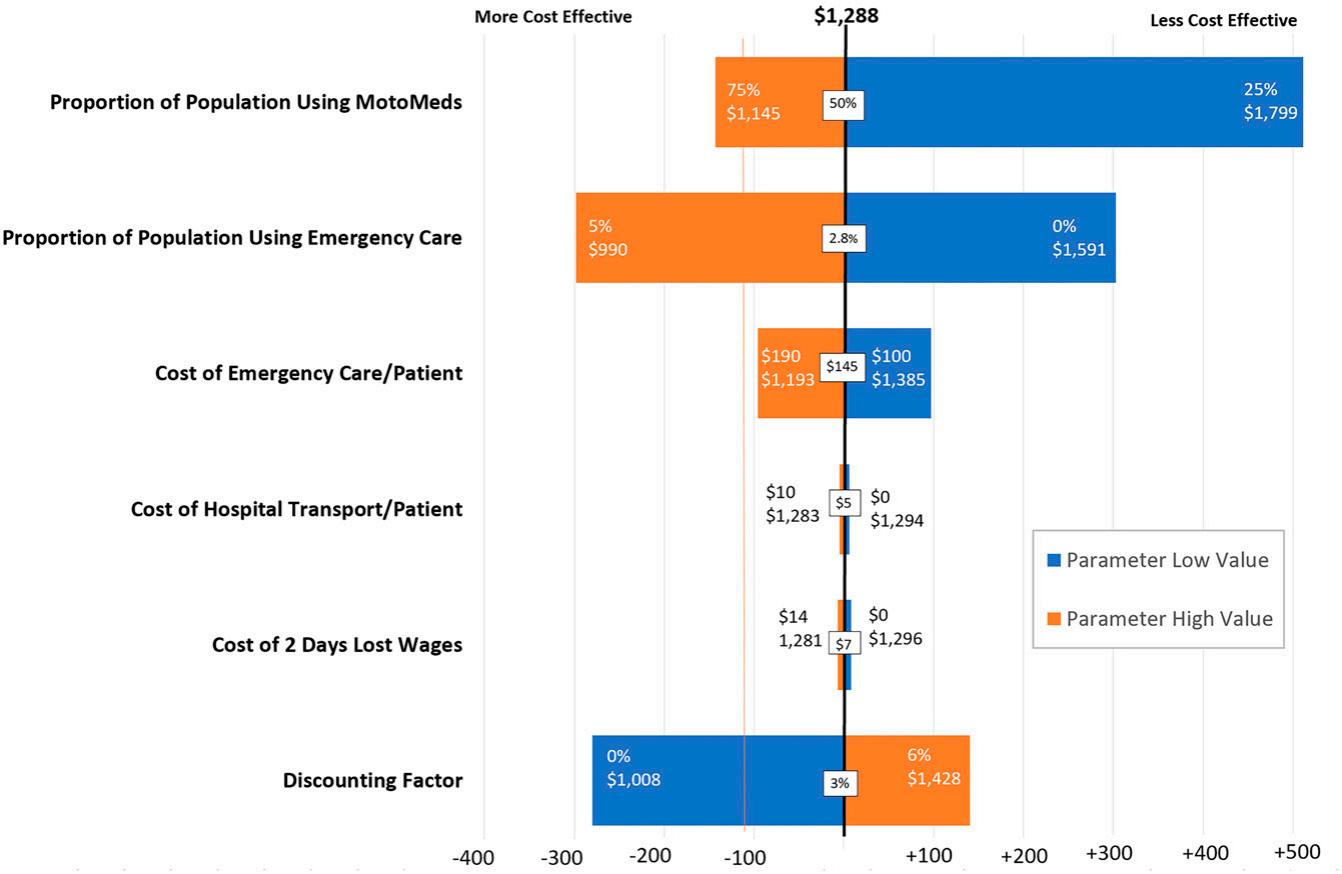
Sensitivity analysis. Univariate sensitivity analysis was performed to evaluate how uncertainty of individual parameter estimates affected the Incremental Cost Effectiveness Ratio in relation to the per capita GDP in Haiti. The vertical orange line designates the per capita GDP of Haiti ($1,177). Two times the per capita GDP is $2,354. Three times the per capita GDP is $3,531 (the cost-effectiveness threshold). This figure appears in color at www.ajtmh.org.

Through sensitivity analysis the incremental cost-effectiveness ratio ranged from $990 to $1,799 (Figure 4). The incremental cost-effectiveness ratio decreased, signifying a higher degree of cost-effectiveness, when the following parameters were increased: telemedicine and medication delivery service utilization, hospital emergency care utilization, cost of hospital emergency care, cost of hospital transport, and daily wages. A decreased discounting factor was likewise associated with a lower incremental cost-effectiveness ratio signifying a higher degree of cost-effectiveness. Through all parameter variations the incremental cost-effectiveness ratio remained within 2× the per capita GDP of Haiti and thus signified cost-effectiveness.

## DISCUSSION

Here, we demonstrated the cost-effectiveness of a nighttime telemedicine and medication delivery service compared with hospital emergency services in Haiti. The telemedicine and medication delivery service has the potential to avert additional years of life lost and years lost to disability among children under 10 for a relatively low cost to society. The service’s greatest costs relate to compensation of drivers and nurses. In this way, finances ideally are funneled directly back into society.

The results were robust to all input variables. Sensitivity analyses showed that the proportion of the population utilizing the service and the proportion of the population utilizing hospital emergency care were the key parameters influencing the incremental cost-effectiveness ratio (Figure 4). Increased telemedicine and medication delivery service utilization and increased hospital emergency care utilization are positively associated with the incremental cost-effectiveness ratio.

While alternative telemedicine and medication delivery service operational modes and scales were not considered, it is important to note that increased service utilization was found to be positively associated with cost-effectiveness. Therefore, a positive feedback loop where professionalism, reasonable payment structure and word-of-mouth perpetuate service usage has the potential to further increase the cost-effectiveness of the service.

This cost-effectiveness analysis may stimulate interest in innovative solutions to improve access to care like the service evaluated herein. Other telemedicine and medication delivery services have been largely limited to HIC ^41^; however, there is likely an underappreciated need for both telemedicine and medication delivery in LMIC. Successful telemedicine use cases include the Aponjon remote consultation service for maternal, neonatal, and infants in Bangladesh that provided valuable medical advice and support but lacked a referral system ^42^ and the Uliza! Clinicians’ HIV Hotline in Kenya that effectively assisted healthcare providers to manage their HIV+ patients. ^43^ Successful medication delivery cases include Riders for Health that champions efficient diagnosis of infectious diseases to prevent progression and spread in seven LMIC. ^21^ The cost-effectiveness analysis herein may bolster support for these initiatives and other services in LMIC.

Weaknesses of this study include failure to obtain the provider/institutional costs of hospital emergency care. It is likely that local hospitals do not have the level of granular data needed and efforts to find it in the literature were unsuccessful. ^35^

Other weaknesses include failure to account for patients with nighttime symptoms progressing at a pace sufficiently slow to seek basic clinical care in the morning. In Haiti, primary clinical care is not available at night and thus was not included as a comparator. However, to evaluate the true cost-effectiveness of the telemedicine and medication delivery service, future studies should consider the proportion of nonprogressive cases and the cost of morning clinical care. Other future analyses should consider the deteriorated state of patients seeking emergency care.

Limitations in data availability likely contributed to a conservatively high incremental cost-effectiveness ratio. The estimate of hospital emergency care usage is based on the INACT-1 study which asked families to recall illnesses from the past month but did not specifically ask which illnesses required pediatric hospital level care; thus the true hospital emergency care usage is likely to be higher. MotoMeds service cares for patients 10 years and under but DALYs in this analysis were calculated for children aged 0–9 years. Additionally, the service averts DALYs caused by other illnesses not included in this analysis.

### Conclusions.

A telemedicine and medication delivery service for nighttime pre-emergency pediatric care is likely to be cost-effective compared with hospital emergency care in semi-urban and rural Haiti. This form of telemedicine and medication delivery service may represent an innovative option to extend access to care to isolated families at night across Haiti and possibly in other LMIC with similar challenges.

## Supplemental Material


Supplemental materials
