# Supplementary material for: A Nighttime Telemedicine and Medication Delivery Service to Avert Pediatric Emergencies in Haiti: An Exploratory Cost-Effectiveness Analysis
Source: Am J Trop Med Hyg. 2022 Feb 21;106(4):1063–71. doi: 10.4269/ajtmh.21-1068 (PMC8991343; doi:10.4269/ajtmh.21-1068)
Supplement: Supplementary file 1 [file tpmd211068.SD1.pdf]

SUPPLEMENTAL MATERIALS

Supplement 1. Estimates of population accessible and covered by MotoMeds

	Population	Accessible Population	Eligible Population (21%)	Population Likely to Use MotoMeds (50%)	Population Likely to Use HEM (2.8%)	MotoMeds Patient Load	Hospital Emergency Care Patient Load
Jacmel	195,674	65,630	13,782	6,891	386	1460	41
Leogane	208,799	173,838	36,506	18,253	1022	2555	71
Gressier	38,092	33,971	7,134	3567	200	6570	184
Total	442,565	273,440	57,422	28711<br>Age 0: 3,008<br>Ages 1 to 4: 10,254<br>Ages 4 to 9: 15,449	1608<br>Age 0: 168<br>Ages 1 to 4: 574<br>Ages 4 to 9: 865	10,585	296

Supplement 2. A. Map indicating extent of panel B. B. Map of MotoMeds service areas in Haiti: Gressier, Leogane, and Jacmel. Blue crosses represent MotoMeds service areas. Geospatial analyses were conducted in ArcGIS 10.7.1; Basemap attributions are watermarked on figure by ArcGIS per ESRI policy[1, 2].

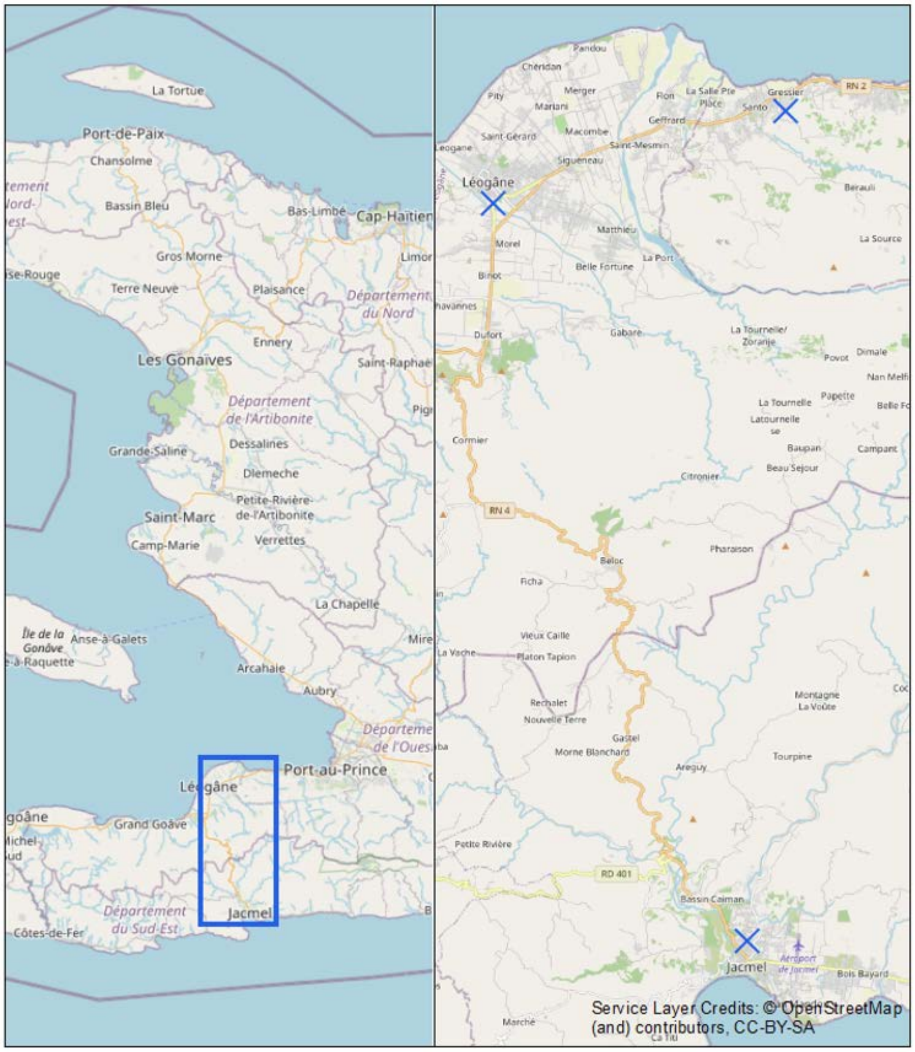

References

1. ESRI, 2019. ArcGIS. Redlands, CA: Environmental Systems Research Institute.
2. Open Street Map Contributors, 2021. Open Street Map.
